# Canine Adrenomedullary and Pheochromocytoma Organoids: A Novel In Vitro Model

**DOI:** 10.1210/endocr/bqaf114

**Published:** 2025-06-27

**Authors:** Marit F van den Berg, Elpetra P M Timmermans-Sprang, Fleur C Viets, Lucas van den Berg, Fatima Danawar, Monique E van Wolferen, Hans S Kooistra, Guy C M Grinwis, Wilhelmina H A de Jong, Martijn van Faassen, Sara Galac

**Affiliations:** Department Clinical Sciences, Faculty of Veterinary Medicine, Utrecht University, Utrecht 3584 CM, The Netherlands; Department Clinical Sciences, Faculty of Veterinary Medicine, Utrecht University, Utrecht 3584 CM, The Netherlands; Department Clinical Sciences, Faculty of Veterinary Medicine, Utrecht University, Utrecht 3584 CM, The Netherlands; Department Clinical Sciences, Faculty of Veterinary Medicine, Utrecht University, Utrecht 3584 CM, The Netherlands; Department Clinical Sciences, Faculty of Veterinary Medicine, Utrecht University, Utrecht 3584 CM, The Netherlands; Department Clinical Sciences, Faculty of Veterinary Medicine, Utrecht University, Utrecht 3584 CM, The Netherlands; Department Clinical Sciences, Faculty of Veterinary Medicine, Utrecht University, Utrecht 3584 CM, The Netherlands; Department Biomolecular Health Sciences, Faculty of Veterinary Medicine, Utrecht University, Utrecht 3584 CL, The Netherlands; Department Clinical Sciences, Faculty of Veterinary Medicine, Utrecht University, Utrecht 3584 CM, The Netherlands; Department of Laboratory Medicine, University of Groningen, University Medical Center Groningen, Groningen 9713 GL, The Netherlands; Department Clinical Sciences, Faculty of Veterinary Medicine, Utrecht University, Utrecht 3584 CM, The Netherlands

**Keywords:** dog, adrenal, adrenal medulla, culture, modeling

## Abstract

**Context:**

Given the lack of effective medical treatment for pheochromocytomas (PCCs), a reliable in vitro model is needed to explore new therapies. Organoids are three-dimensional (3D) self-renewing structures that exhibit key features of their tissue of origin, providing valuable platforms for disease modeling and drug screening.

**Objective:**

This study aimed to establish and characterize organoid cultures of canine normal adrenal medullas and PCCs.

**Methods:**

Normal adrenal medullas from healthy dogs and tumor tissue from client-owned dogs with PCC were used to develop organoids. Primary cell suspensions were cultured in a 3D matrix, and organoids were established under optimized conditions. Organoids were characterized using histology, immunohistochemistry, immunofluorescence, qPCR, and metanephrine analysis by LC-MS/MS.

**Results:**

Five adrenomedullary organoid lines were successfully established, demonstrating sustained growth. Organoid cultures were also derived from 9 PCCs, although expansion was limited after passages 1 to 2. Both adrenomedullary and PCC organoids expressed differentiation markers (chromogranin A, synaptophysin, phenylethanolamine N-methyltransferase) and stem/progenitor markers (nestin, SOX10). Organoids retained key functional traits, as indicated by metanephrine levels in culture supernatants, which initially mirrored primary tumor patterns. A decline in both differentiation marker expression and metanephrine levels was observed over time, possibly due to organoid dedifferentiation or selective loss of differentiated chromaffin cells.

**Conclusion:**

This study demonstrates the establishment of the first adrenomedullary and PCC organoid lines. While further optimization is needed, these organoids offer valuable potential as an in vitro model to investigate PCC pathophysiology and explore novel treatment strategies for this therapeutically challenging tumor.

Pheochromocytomas (PCCs) are rare neuroendocrine tumors arising from chromaffin cells in the adrenal medulla. In humans, PCCs typically present with symptoms of catecholamine excess ranging from mild manifestations, such as palpitations, sweating, and headaches, to more severe, life-threatening cardiovascular complications, like arrhythmias, myocardial infarction, and hypertensive crisis (1). In addition to these symptoms, clinical signs may arise from the mass effect or invasive nature of the tumor. Surgical removal is the therapy of choice, but may be precluded by extensive invasion, distant metastasis, or severe concurrent disease. For these patients, medical therapy is warranted. The lack of suitable experimental models has hindered preclinical drug development, thus limiting the availability of appropriate treatment options for patients (2, 3). This limitation is particularly critical for metastatic PCC, which carries a poor prognosis, with a median overall survival of approximately 7 years, and for which no highly effective treatment options currently exist (4, 5).

Existing experimental models often fail to reflect the full genetic and phenotypic complexity of these tumors, underscoring the need for more representative alternatives. While progress has been made with succinate dehydrogenase complex subunit B (*SDHB*)-deficient mouse and rat models, these models rely on radiation or genetic modifications to induce tumor development, which contrasts with the spontaneous tumor formation seen in human disease. Moreover, they face substantial interspecies differences (2, 3). Additionally, they represent only a single genotype, whereas more than 20 susceptibility genes have been implicated in human PCCs and paragangliomas (6).

Organoids are self-organizing, self-renewing three-dimensional (3D) cellular structures derived from stem cells that contain organ-specific cell types. Compared to traditional two-dimensional cell cultures, they better preserve key aspects of cellular organization, differentiation, and functionality, making them a valuable platform for disease modeling and drug screening (7). To date, however, no organoid models of PCCs have been successfully established in any species. Interestingly, PCCs occur naturally in dogs and share clinical, biochemical, histological, and genetic similarities with human PCCs (8-10). Combined with the opportunities offered by the advanced level of medical care available to veterinary patients today, this makes the dog a highly relevant animal model for both in vitro research and translational in vivo studies, potentially overcoming the limitations of current models.

The aim of this study was to establish and characterize canine adrenomedullary and PCC organoids. By generating these organoid lines, we aim to create a robust platform that can ultimately serve as a research tool to investigate the pathophysiology of PCCs and explore potential therapeutic approaches.

## Methods

### Patient Population and Sample Collection

Tumor tissue was obtained from 9 client-owned dogs with biochemically confirmed PCC following surgical removal. Biochemical confirmation was based on elevated plasma metanephrine and normetanephrine concentrations (11). These dogs presented with various clinical signs, including polyuria/polydipsia, panting, and hypertension. Additionally, 2 dogs had acute hemoabdomen, while 3 cases were diagnosed with PCC following an endocrine workup for an incidentally detected adrenal mass. Clinicopathological data of these dogs are provided in Table 1. Tumor tissues were cut longitudinally, with half of the mass being formalin-fixed and paraffin-embedded for histopathology and immunohistochemistry (IHC). In all dogs, the diagnosis of PCC was confirmed by histopathology and IHC using chromogranin A (CHGA) and synaptophysin (SYP). A representative piece of the tumor, excluding necrotic or hemorrhagic areas, was snap-frozen in liquid nitrogen and then stored at −70 °C until RNA extraction. The remainder of the mass was separated from the adrenal cortex and capsule, transferred to a petri dish on ice containing Dulbecco's Modified Eagle Medium (DMEM)/F-12 (Gibco, New York, USA) with 1% penicillin/streptomycin (P/S; 10 000 U/mL; Gibco) and 10% fetal calf serum (Gibco), and cut into small fragments of 2 to 3 mm^3^. These fragments were either frozen in Recovery Cell Culture Freezing Medium (Gibco) using a Mr. Frosty freezing container (Nalgene, NY, USA) and stored in liquid nitrogen (−196 °C), or directly processed for organoid culture.

**Table 1.** Clinical and pathological data of the 9 dogs from which PCC organoid cultures were established

PCC	Breed	Age (y)	Sex	pNMN (nmol/L)	pMN (nmol/L)	Metastasis	Ki67 (%)	Location	Tumor size (cm)
1	Galgo Espanol*^a^*	11.4	FN	>10.97	1.63	No	0.05	Left adrenal	5.1
2	Stabyhoun	8.9	MN	11.89	2.06	No	6.1	Left adrenal	2.3
3	Kooiker	13.7	MN	5.07	1.03	No	11.4	Right adrenal	2.0
4	Rhodesian Ridgeback	9.5	MN	35.76	2.51	No	5.2	Right adrenal	3.5
5	Australian cattle dog	13.3	FN	19.52	9.9	Likely*^b^*	12.5	Left adrenal	3.0
6	Basenji	9.7	FE	4.92	9.54	No	6.9	Left adrenal	3.2
7	Mixed breed	14.0	MN	26.37	3.29	No	7.5	Left adrenal	2.7
8	Schnauzer*^a^*	10.6	FN	85.02	>50.99	Possibly*^c^*	10.4	Left adrenal	5.5
9	Mixed breed	12.7	FN	7.19	1.91	No	0.5	Right adrenal	1.1

The upper reference limits for plasma normetanephrine and metanephrine concentrations in dogs are 3.56 nmol/L and 2.49 nmol/L, respectively, as previously reported (11). Staging was performed by contrast-enhanced abdominal and thoracic CT, with targeted sampling (fine-needle aspiration and histological examination) as indicated. Tumor location and tumor size were determined based on diagnostic imaging findings.

Abbreviations: FE, female entire; FN, female neutered; MN, male neutered; pNMN, plasma normetanephrine; pMN, plasma metanephrine; y, years.

^*a*^Presented with hemoabdomen.

^*b*^Pulmonary nodules and cranial mediastinal and abdominal lymphadenopathy raised suspicion for metastasis, but cytology or histology were not performed.

^*c*^Enlarged sternal and jejunal lymph nodes were identified but were not sampled, so metastatic disease could not be definitively ruled out.

Normal adrenal tissues were obtained from healthy dogs used in animal research, euthanized for reasons unrelated to the present study, which was approved by the Ethical Committee of the Faculty of Veterinary Medicine, Utrecht University. Clinical data of these dogs are provided in Supplementary Table S1 (12). Adrenal glands were cut longitudinally, and medullas were separated from the adrenal cortices. Adrenal medullas were pooled from 3 or 4 dogs to obtain sufficient tissue. These medullary tissues were immediately processed for organoid culture using the same protocol applied to the tumor samples, including preparation in DMEM/F-12 medium supplemented with 1% P/S and 10% fetal calf serum, followed by fragmentation and culture initiation.

### Organoid Establishment

**Figure 1.**
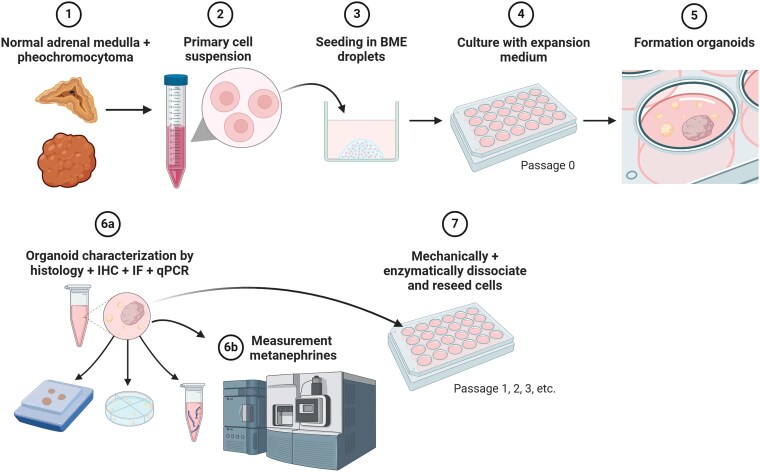
Schematic representation of adrenomedullary and pheochromocytoma organoid formation and characterization. Tissues are dissociated into single-cell suspensions, embedded in basement membrane extract (BME), and seeded into droplets. Expansion medium is applied and refreshed regularly until organoids form. Organoids undergo characterization through histology, immunohistochemistry (IHC), immunofluorescence (IF), and quantitative real-time RT-PCR (qPCR). Metanephrine secretion is quantified in cell culture supernatants using liquid chromatography-tandem mass spectrometry (LC-MS/MS). Organoid cultures are maintained and expanded through passaging, which involves mechanical and enzymatic dissociation of the cellular structures. Created in BioRender. Galac, S. (2025) https://BioRender.com/jn7aotu.

Tissue fragments from normal adrenal medullas (NAMs) and PCCs were mechanically and enzymatically dissociated to generate single cells, followed by red blood cell depletion. After counting, cell suspensions were mixed with cold Cultrex® basement membrane extract (BME; R&D systems, Minneapolis, MN, USA) in a 1:3 volume ratio to create 15 μL droplets on prewarmed 48-well plates. Following gelation, prewarmed expansion medium (EM) was added to the solidified droplets. Cells were cultured at 5% CO_2°_ and 37 °C. For a schematic overview of the organoid generation and characterization process, see Fig. 1. For full details on mechanical and enzymatic dissociation steps, red blood cell lysis, medium composition, and microscopic evaluation of organoid cultures, see Supplementary File S1 and Table S2 (12).

### Organoid Expansion

The EM consisted of Advanced DMEM/F12 medium supplemented with 1% P/S, 1% GlutaMAX supplement (Gibco), 10 mM HEPES (Gibco), 2% B27 supplement without vitamin A (Gibco), 1% N2 supplement (Gibco), 1 mM N-acetylcysteine (NAC; Sigma-Aldrich, Merck KGaA, Darmstadt, Germany), and 2 mM calcium gluconate (Sigma-Aldrich), optimized as detailed in Table S2 (12). Primocin (InvivoGen, San Diego, CA, USA) and Y-27632 dihydrochloride (AbMole BioScience, Houston, USA) was added once to the newly established organoid culture.

For optimal organoid growth, a combination of growth factors was incorporated into the EM, referred to as WREFLD (Wnt, R-spondin-3, EGF, FGF2, LIF, DHEAS). This combination was optimized as detailed in Table S2 (12). The selected concentrations of each factor were: 0.25 nM Wnt surrogate (ImmunoPrecise Antibodies, Utrecht, The Netherlands), 2% R-spondin-3 (ImmunoPrecise Antibodies), 20 ng/mL epidermal growth factor (EGF; Peprotech, Cranbury, NJ, USA), 20 ng/mL fibroblast growth factor 2 (FGF2; Peprotech), 20 ng/mL recombinant human leukemia inhibitory factor (LIF; Peprotech), 2 μM dehydroepiandrosterone sulfate (DHEAS; Cerilliant, Texas, USA). The EM, including WREFLD, was refreshed 2 to 3 times per week by removing half of the supernatant volume and replacing it with fresh EM.

### Passaging of Organoid Cultures

To assess the long-term self-renewing potential of organoids, organoids and dense cellular clusters were dissociated, combined, and reseeded for subsequent passages at a ratio of 1:1.5 to 1:2 (ie, for each well, 1.5 to 2 new wells were seeded with the cell suspension during passaging). After gelation, prewarmed medium—comprising 40% conditioned medium from the previous passage (to preserve secreted factors) and 60% fresh EM supplemented with WREFLD and 20 µM Y-27632 dihydrochloride—was added. For the complete step-by-step protocol, see Supplementary File 1 (12).

### Cryopreservation of Organoids

Dense cellular structures were enzymatically and mechanically dissociated, while organoids were mechanically fragmented. The resulting suspensions were pooled, washed, and resuspended in cold Recovery Cell Culture Freezing Medium. Samples were then frozen using a Mr. Frosty freezing container at −70 °C and stored in liquid nitrogen (−196 °C). A detailed protocol is provided in Supplementary File 1 (12).

### Organoid Differentiation

For differentiation, PCC organoid cultures were treated with 10 µM dexamethasone (Dex; Sigma-Aldrich) and/or 100 nM phorbol 12-myristate 13-acetate (PMA; Millipore, Billerica, MA, USA), with or without the growth factor combination WREFLD.

### Metanephrine Measurements

Hormone measurements were performed in the conditioned medium collected from PCC organoid cultures at multiple time points to study the functional properties of PCC organoids. Metanephrine, normetanephrine, and 3-methoxytyramine concentrations were quantified using liquid chromatography–tandem mass spectrometry (LC-MS/MS), as previously described and validated for human and canine samples (11, 13). Metanephrines were measured instead of catecholamines due to their greater stability, continuous production independent of active secretion, and superior diagnostic sensitivity and specificity for PCC (14). All measurements were performed in duplicate, with each duplicate consisting of a pooled sample from 6 wells. Due to the long-term nature of the culturing process, cells were not lysed for protein quantification, and thus normalization to total protein content was not performed. Instead, the number of wells from which conditioned medium was collected was standardized across all samples. A negative control consisted of culture medium with added growth factors. Immediately after processing, all samples were stored at −70 °C (for a maximum of 11 months) before being transported on dry ice to the University Medical Center Groningen. The samples were stored at −80 °C until analysis, which was performed within 2 months of receipt.

### Selection of Markers for Characterization of Organoids

Based on previous studies, we have selected a range of markers to characterize the cellular composition of organoids. These markers include SRY-box transcription factor 2, 9, and 10 (SOX2, SOX9, and SOX10), vimentin (VIM), and nestin (NES), which are commonly used to identify stem/progenitor cells within the adrenal medulla (15-19). While S100 calcium-binding protein B (S100B) and glial fibrillary acidic protein (GFAP) are primarily used to identify sustentacular cells, we also used them to examine the potential progenitor role of these cells (17, 18). Additionally, we focused on CHGA, SYP, and phenylethanolamine N-methyltransferase (PNMT) as chromaffin cell markers, and tubulin beta 3 class III (TUBB3) and microtubule-associated protein 2 (MAP2) as neural markers (20). Tyrosine hydroxylase (TH) served as a combined marker for both chromaffin cells and catecholaminergic neurons. Finally, Ki67 was used as a marker for proliferation.

### Histology and Immunohistochemistry

Primary tumors, organoids, and dense cellular clusters were fixed in 4% w/v buffered paraformaldehyde, embedded in paraffin, and sectioned at 4 μm. For IHC, slides underwent antigen retrieval, blocking, and subsequent incubation with primary antibodies followed by HRP-conjugated secondary antibodies. Slides were then counterstained with hematoxylin. Positive control tissues were used for each antibody, and omission of the primary antibody from the immunohistochemical protocol served as a negative control. For full details on fixation, embedding, and staining, see Table 2 and Supplementary File 1 (12).

**Table 2.** List of antibodies used in IHC analyses

Antibody	Host	Research Resource Identifier	Antigen retrieval	Dilution
CHGA	Mouse	Thermo Fisher Scientific Cat# MA5-13096, RRID:AB_10987033	Citrate, 98 °C, 60 minutes	1:100
SYP	Mouse	Agilent Cat# M7315, RRID:AB_2687942	Citrate, 98 °C, 30 minutes	1:100
NES	Rabbit	Thermo Fisher Scientific Cat# PA5-11887, RRID:AB_2148923	Citrate, 98 °C, 30 minutes	1:500
VIM	Mouse	BioGenex Cat# AM074GP, RRID:AB_3101770	Citrate, 98 °C, 60 minutes	1:500
SOX10	Mouse	Santa Cruz Biotechnology Cat# sc-365692, RRID:AB_10844002	Tris-EDTA, 98 °C, 30 minutes	1:500
Ki67	Mouse	Agilent Cat# M7240, RRID:AB_2142367	Tris-EDTA, 98 °C, 30 minutes	1:200
Anti-mouse IgG	Goat	ImmunoLogic Cat# DPVM110HRP, RRID:AB_2915957		
Anti-rabbit IgG	Goat	ImmunoLogic Cat# DPVR110HRP, RRID:AB_2915958		

Abbreviations: CHGA, chromogranin A; IHC, immunohistochemistry; NES, nestin; SOX10, SRY-box transcription factor 10; SYP, synaptophysin; VIM, vimentin.

### Immunofluorescence Staining of Organoids

Cell suspensions in BME were seeded as droplets on prewarmed 4-compartment CellView™ cell culture dishes (Greiner Bio One, Frickenhausen, Germany) or prewarmed CellView™ slide 10-well chamber slides (Greiner Bio One) and cultured until organoids formed. Organoids were fixed in 3% paraformaldehyde with 0.1% glutaraldehyde, then quenched, permeabilized, and blocked. Samples were incubated with primary antibodies followed by fluorophore-conjugated secondary antibodies (Table 3), counterstained with 4′,6-diamidino-2-phenylindole (DAPI), and mounted. For the complete step-by-step protocol, see Table 3 and Supplementary File 1 (12).

**Table 3.** List of antibodies used in IF analyses

Antibody	Host	Research Resource Identifier	Dilution
CHGA	Mouse	Thermo Fisher Scientific Cat# MA5-13096, RRID:AB_10987033	1:100
SYP	Mouse	Agilent Cat# M7315, RRID:AB_2687942	1:100
PNMT	Rabbit	Abcam Cat# ab154282, RRID:AB_3676368	1:100
TH	Rabbit	Novus Cat# NB300-109, RRID:AB_10077691	1:1000
TUBB3	Chicken	Sigma-Aldrich Cat# AB9354, RRID:AB_570918	1:1000
NES	Rabbit	Thermo Fisher Scientific Cat# PA5-11887, RRID:AB_2148923	1:300
VIM	Mouse	BioGenex Cat# AM074GP, RRID:AB_3101770	1:80
Anti-rabbit IgG Alexa Fluor™ 488	Goat	Thermo Fisher Scientific Cat# A-11008, RRID:AB_143165	1:1000
Anti-chicken FITC	Goat	Thermo Fisher Scientific Cat# A16055, RRID:AB_2534728	1:1000
Anti-mouse IgG Alexa Fluor™ 568	Goat	Thermo Fisher Scientific Cat# A-11004, RRID:AB_2534072	1:1000

Abbreviations: CHGA, chromogranin A; IF, immunofluorescence; NES, nestin; PNMT, phenylethanolamine N-methyltransferase; SYP, synaptophysin; TH, tyrosine hydroxylase; TUBB3, tubulin beta-3; VIM, vimentin.

### Image Acquisition

Fluorescence images were acquired using an Olympus SpinSR10 spinning disk confocal microscope equipped with a 30× silicone-oil immersion objective and ORCA fusion sCMOS cameras. Organoids were imaged as single mid-plane slices or as Z-stacks, using standardized settings for lasers, filters, exposure times, and Z-step increments. For full acquisition parameters, see Supplementary File 1 (12).

### Image Processing and Analysis

Fluorescence images were processed in FIJI (version 1.54f) (21) using a custom macro to define regions of interest (ROIs), extract fluorescence intensity data, and export results. Data were analyzed in GraphPad Prism (version 10.1.1, GraphPad Software Inc., Boston, MA, USA), with each sample's mean intensity normalized to its corresponding negative control (organoids stained with secondary antibodies only) to calculate fold-over-background. For complete details, see Supplementary File 1 (12).

### RNA Isolation and Quantitative Real-time RT-PCR

RNA was isolated from primary (tumor) tissue, cell suspensions, and organoids and dense cellular clusters at different passages, and RNA concentrations were measured. cDNA was synthesized, and quantitative real-time RT-PCR (qPCR) analyses were performed to determine the expression levels of adrenomedullary markers *CHGA*, *SYP*, *TH*, and *PNMT*, stem/progenitor markers *NES*, *VIM*, *SOX2*, *SOX9*, *S100B*, and *GFAP*, and neural markers *TUBB3,* and *MAP2*. Reference genes were selected via geNorm method (22), and relative expression was calculated using the 2^−ΔΔCt method (23). For complete details on RNA handling, primer design and validation, and reference gene selection, see Supplementary File 1 and Table S3 (12).

### Statistical Analysis

Statistical analyses were performed using GraphPad Prism software (version 10.1.1; GraphPad Software Inc. Dotmatics, Boston, MA, USA). Data were first assessed for normality using the Shapiro-Wilk test. For comparisons among multiple independent groups, the Kruskal-Wallis test was applied for non-normally distributed data, while one-way ANOVA was used for normally distributed data. For matched datasets (eg, gene expression levels in PCC tissues, cell suspensions, and organoids derived from the same PCC), Friedman's test was performed. When significant differences were observed, post hoc analyses were conducted with Dunn's correction for multiple comparisons. A *P* value < .05 was considered statistically significant.

## Results

### Generation of Canine Adrenomedullary and Pheochromocytoma Organoids

Five adrenomedullary organoid lines, derived from NAMs of healthy dogs, were successfully established. Additionally, 9 PCC organoid lines were established from tumor tissues obtained from client-owned dogs following adrenalectomy.

**Figure 2.**
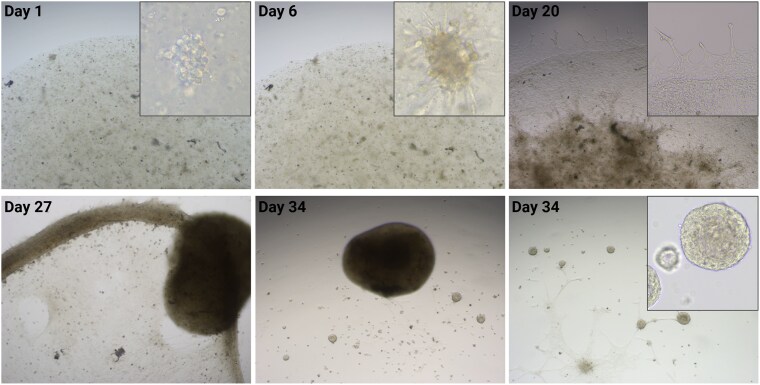
Representative bright-field images of PCC9 in P0, showing the development of organoids from the initial plating of a cell suspension in a BME droplet through to passaging. On day 1, single chromaffin cells and small cell aggregates are visible within the BME droplet. By one week, cell clusters have increased in size, and some cells exhibit elongated, varicose extensions. Outside the BME droplet, migrating cells flatten and extend varicose processes (day 20). Over time, within the BME droplet, the clusters expand, densify, and interconnect to form a network that gradually contracts into a dense, compact cellular cluster (day 27). Simultaneously, small organoids emerge outside the BME droplet and at the site where the droplet detached from the bottom of the well, increasing in size and number. At later stages, both organoids and the dense cellular cluster are evident (day 34). Images were taken at 4× magnification, with insets at 20× for detailed visualization.

**Figure 3.**
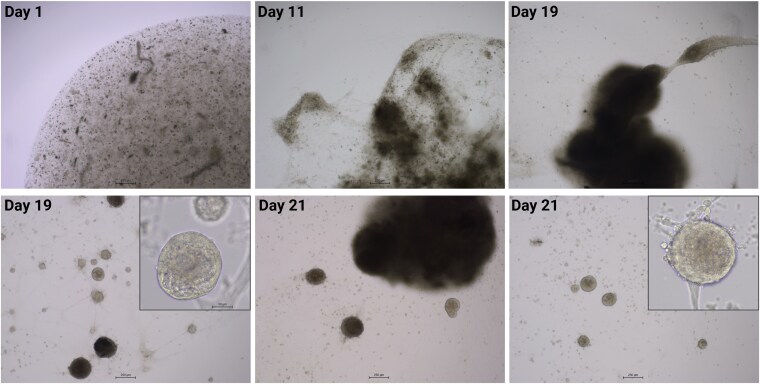
Representative bright-field images of NAM4 in P0 show the more rapid progression of organoid development compared to PCC organoid cultures. By day 11, cell clusters have formed and begun contracting into dense structures. By day 19, these clusters have expanded and interconnected, forming dense cellular clusters. By day 21, just before passaging, numerous organoids have formed. Images were taken at 4× magnification (scale bar: 250 µm), with insets at 20× for detailed visualization.

The organoid cultures exhibited distinct developmental stages. Initially, after seeding the cell suspension, cells were present as single cells or small aggregates and displayed a chromaffin-like morphology, characterized by a rounded shape with sizes typically ranging between 5 and 15 µm. Occasionally, small granules were visible within the cells. After approximately one week, cells began to form clusters within the BME droplet. Over time, these clusters became denser as cells appeared to contract toward each other. Meanwhile, some cells underwent morphological changes: some acquired an elongated morphology, resembling a mesenchymal phenotype, while others extended long, varicose processes resembling neural structures such as axons or dendrites. As this process continued, the clusters interconnected, forming a larger cellular network. This network gradually condensed into a dense, compact cellular structure that eventually detached from the bottom of the well. At the same time, some cells migrated outside the BME droplet, attached to the bottom of the well, and began to flatten out and extend processes. Over time, the number of these cells increased, and they formed three-dimensional spheroid-like structures, referred to as organoids, which gradually increased in both number and size. While the dense cellular clusters formed as large cell aggregates contracted and merged within the BME droplet (or its remnants), the organoids gradually developed outside of it. Figures 2 and 3 show the progression through all stages of organoid development for both PCC and adrenomedullary organoids cultures. Adrenomedullary organoid cultures progressed through these stages more rapidly compared to PCC organoid cultures. In passage 0 (P0), organoid formation for NAMs typically took 14 to 27 days, whereas PCC organoid cultures required a longer timeframe of 19 to 72 days (Table 4).

**Table 4.** Total culture duration, highest passage achieved, and time intervals between passage transitions are provided for adrenomedullary and PCC organoid cultures

	Time in culture (days)	Highest passage	P0 > P1 (days)	P1 > P2 (days)	P2 > P3 (days)	P3 > P4 (days)	P4 > P5 (days)	Reason to end culture
NAM1	129	3	14	28-49	10-37			Bacterial infection
NAM2	125	5	18	28-49	10-30	11-37	9	Bacterial infection
NAM3	66	3	15	13	31			Bacterial infection
NAM4	64	2	22	21				Bacterial infection
NAM5	57	2	27	30				Cryopreserved due to fungal infection
PCC1	186	2	26	107				Fungal infection
PCC2	163	2	19	45				Lack of proliferation
PCC3	210	3	31	30	71			Lack of proliferation
PCC4	218	3	47	56	64			Lack of proliferation
PCC5*^a^*	142	1	72					Lack of proliferation
PCC6	199	2	26	63				In culture until the end of the study
PCC7	105	2	43	42				Bacterial infection
PCC8	86	3	51	35				Cryopreserved due to fungal infection
PCC9	41	1	29					Bacterial infection

Abbreviations: NAM, normal adrenal medulla; P, passage; PCC, pheochromocytoma.

^*a*^Stored in liquid nitrogen.

Microscopically, the organoids appeared roughly spherical in shape, with a solid appearance and sometimes a thickened wall. Some organoids exhibited varicose extensions, occasionally forming connections between 2 or more organoids (Figs. 2 and 3).

### Self-Renewal Capacity and Characterization of Pheochromocytoma and Adrenomedullary Organoids

**Figure 4.**
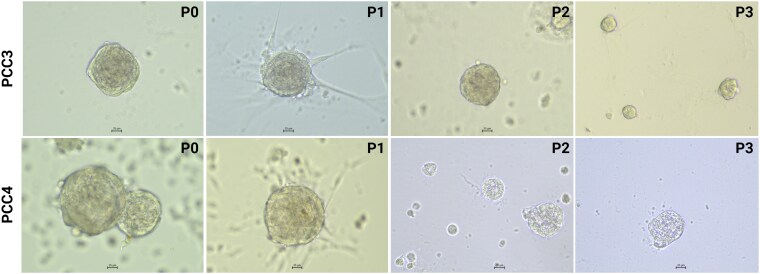
Bright-field images of canine PCC organoids derived from 2 PCCs (PCC3 and PCC4) across 4 consecutive passages (P0, P1, P2, and P3). Scale bars (where shown), 25 μm.

Abbreviations: P, passage; PCC, pheochromocytoma.

**Figure 5.**
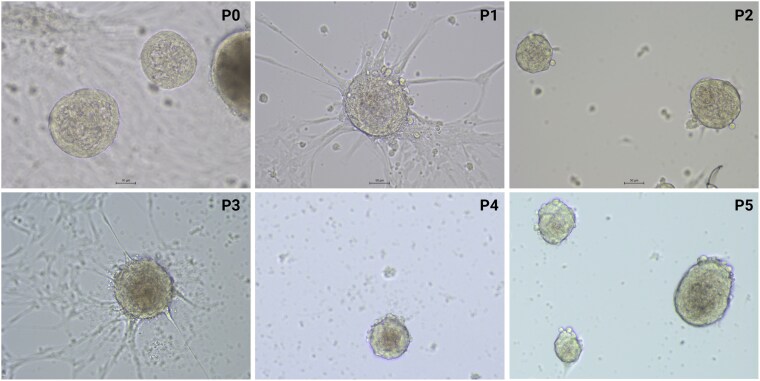
Bright-field images of canine adrenomedullary organoids across 6 consecutive passages (P0-P5). Images were taken at the same magnification. Scale bars (where shown), 50 μm.

Abbreviation: P, passage.

The self-renewal capacity of the organoids was demonstrated by serial passaging, where organoids continued to grow and maintain structural integrity over multiple passages. However, for most PCC cultures, limited self-renewal was observed, as organoid formation and growth decreased after passage 1 or 2. While initial cultures (P0 and P1) exhibited robust organoid formation, the expansion rate decreased in later passages, with longer intervals between passaging. In contrast, adrenomedullary organoids demonstrated more robust self-renewal, reaching higher passage numbers with shorter intervals between passaging. Table 4 summarizes the culture details, including total culture duration, passage intervals, and the highest passage number achieved. Representative bright-field images of PCC organoids at various passages (P0-P3) are shown in Fig. 4, and those of adrenomedullary organoids (at P0-P5) are presented in Fig. 5.

In addition to assessing the self-renewal capacity, we evaluated whether cryopreserved organoids could successfully re-establish growth and morphology. PCC8 organoids from P1 were cryopreserved, thawed, and subsequently cultured. The organoids successfully resumed growth and maintained their morphology, confirming the effectiveness of the cryopreservation protocol.

**Figure 6.**
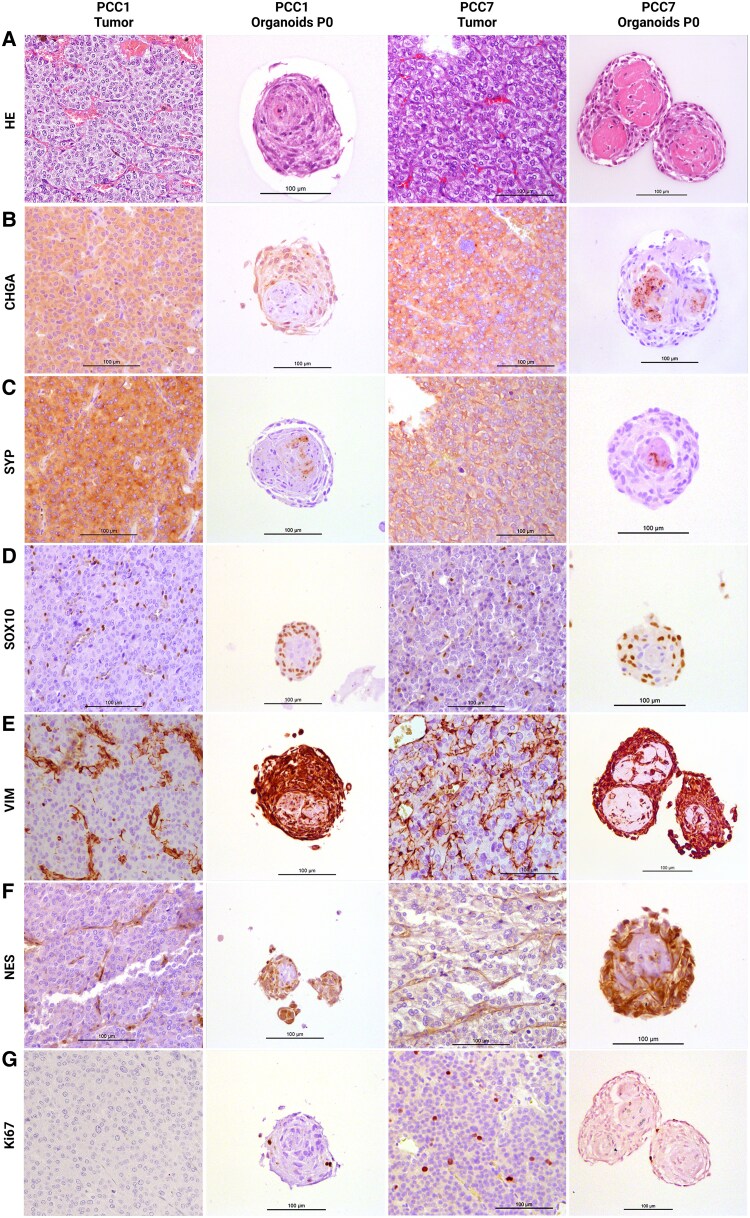
Histology (A) and IHC staining for CHGA (B), SYP (C), SOX10 (D), VIM (E), NES (F), and Ki67 (G) in 2 primary tumors (PCC1 and PCC7) and their derived organoid lines at the end of passage 0. Scale bars (where shown), 100 μm.

Abbreviations: CHGA, chromogranin A; HE, hematoxylin and eosin; NES, nestin; P, passage; PCC, pheochromocytoma; SOX10, SRY-box transcription factor 10; SYP, synaptophysin; VIM, vimentin.

**Figure 7.**
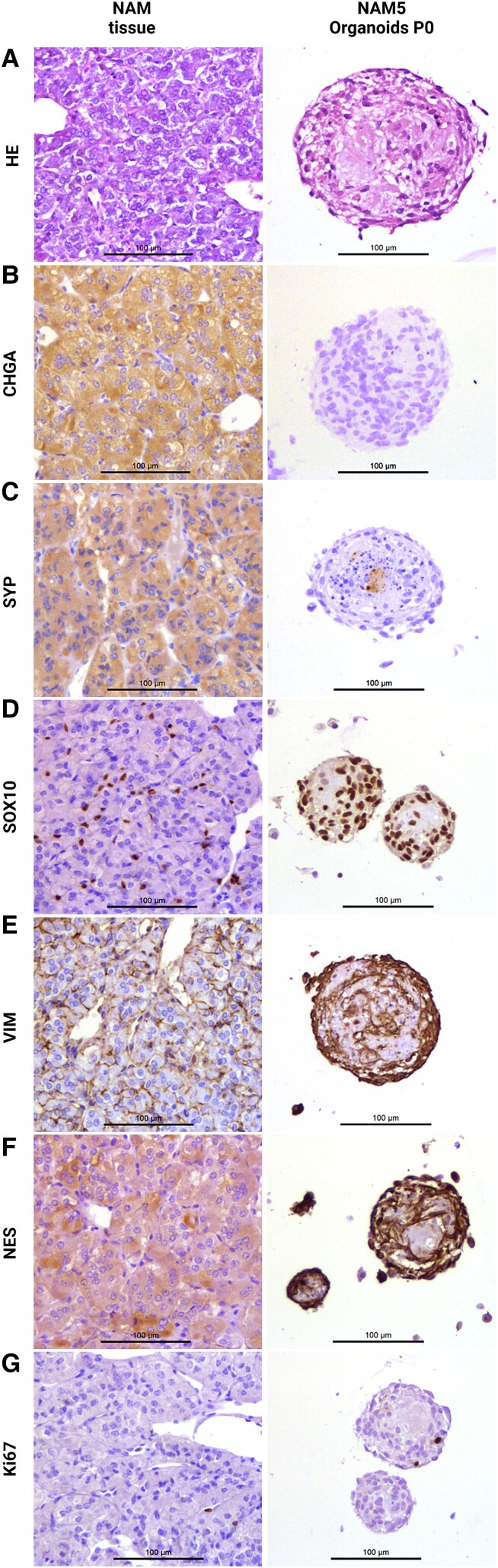
Histology (A) and IHC staining for CHGA (B), SYP (C), SOX10 (D), VIM (E), NES (F), and Ki67 (G) of normal adrenomedullary tissue and adrenomedullary organoids (derived from NAM5) at the end of passage 0. Scale bars (where shown), 100 μm.

Abbreviations: CHGA, chromogranin A; HE, hematoxylin and eosin; NES, nestin; NAM, normal adrenal medulla; P, passage; SOX10, SRY-box transcription factor 10; SYP, synaptophysin; VIM, vimentin.

Histologically, adrenomedullary and PCC organoids exhibited a well-organized structure, with larger organoids frequently containing a central region with eosinophilic material and occasional interspersed cells, surrounded by multiple peripheral cell layers, typically of somewhat elongated cells. In contrast, smaller organoids lacked a distinct central region, exhibiting a more uniform cellular distribution throughout, without the acellular eosinophilic center seen in the larger organoids (Figs. 6A and 7A). Dense cellular clusters, on the other hand, consisted of cells embedded within an extracellular matrix, surrounded by a distinct layer of outer cells. Histological and extracellular matrix staining of PCC and adrenomedullary dense cellular clusters and organoids is shown in Fig. S1 (12). With some frequency, signs of cellular degeneration—characterized by areas suggestive of apoptosis or necrosis (with features such as cytoplasmic eosinophilia, karyopyknosis, and karyorrhexis)—were observed primarily in the central regions of both larger organoids and dense cellular clusters (Fig. S2 (12)). Furthermore, cells displaying morphological characteristics of macrophages were noted occasionally in these central areas, sometimes containing intracytoplasmic deposits reminiscent of hemosiderin pigment.

### Characterization of Organoids Reveals Both Differentiation Marker Expression and Stem/Progenitor Cell Properties

Immunohistochemical analysis revealed the expression of both differentiation and stem/progenitor cell markers within the PCC organoids (Fig. 6). Both SYP and CHGA, markers of differentiated chromaffin cells, showed weaker staining in organoids compared to the primary tumor tissue (Fig. 6B and 6C). Synaptophysin staining exhibited substantial variability, both between organoids from different tumors and among organoids derived from the same tumor. While some organoids were entirely negative for SYP, others contained scattered positive cells. A similar pattern was observed for CHGA. Expression of the stem/progenitor markers NES, VIM, and SOX10 was predominantly observed in the peripheral regions of the organoids, although occasional expression was noted in more central areas (Figs. 6D–6F). SOX10 expression showed considerable variability, with some organoids displaying many positive nuclei, while others had few or none. This variability was observed both between different organoid lines and within the same line. In contrast, all organoids stained consistently positive for NES and VIM. Ki-67 staining indicated occasional proliferative cells, predominantly located in the outer layers of the organoids (Fig. 6G). Immunohistochemical analysis of the dense cellular clusters is presented in the supplementary data (Supplementary Fig. S3 (12)). In the central regions, where extracellular matrix was more abundant, the immunohistochemical staining exhibited a granular pattern that did not clearly associate with cells, and was therefore interpreted as nonspecific. In contrast, distinct cytoplasmic staining was observed in specific cells—typically located in more peripheral regions—indicating the specific expression of the marker (Supplementary Fig. S4 (12)).

Immunohistochemical analysis of an adrenomedullary organoid line revealed an overall staining pattern comparable to that observed in PCC organoids (Fig. 7). Adrenomedullary organoids displayed variable SYP expression (Fig. 7C), which was markedly lower compared to NAM tissue, while VIM and NES staining was consistently present (Fig. 7E and 7F). In contrast to PCC organoids, adrenomedullary organoids were negative for CHGA (Fig. 7B) but showed consistent SOX10 staining (Fig. 7D), with most nuclei being positive. Immunohistochemical characterization of the adrenomedullary dense cellular clusters is provided in the supplementary data (Supplementary Fig. S5 (12)).

**Figure 8.**
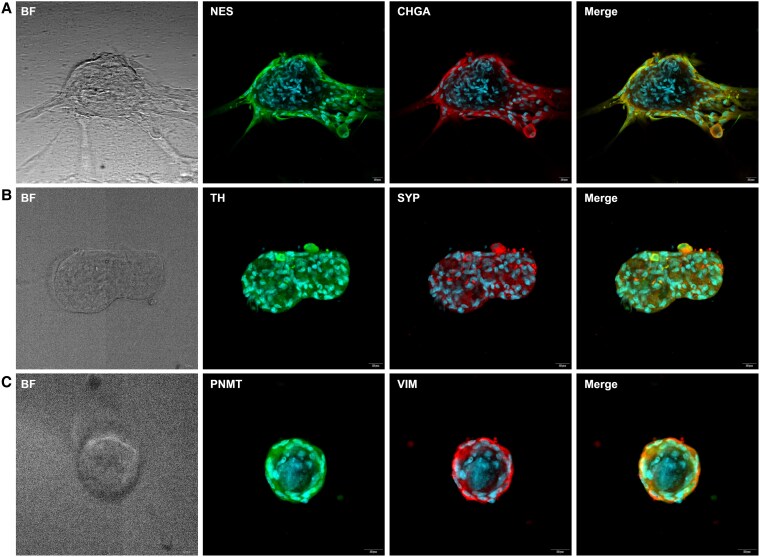
Representative immunofluorescence images of organoids derived from different PCCs, showing staining for differentiated chromaffin cell markers and progenitor/stem cell markers: NES and CHGA (A), TH and SYP (B), and PNMT and VIM (C). The specific markers are indicated at the top of each panel. Alexa Fluor 488- and Alexa Fluor 568-conjugated antibodies were used for detection, and nuclei were counterstained with DAPI. Scale bars represent 20 μm.

Abbreviations: BF, bright field; CHGA, chromogranin A; NES, nestin; PNMT, phenylethanolamine N-methyltransferase; SYP, synaptophysin; TH, tyrosine hydroxylase; VIM, vimentin.

**Figure 9.**
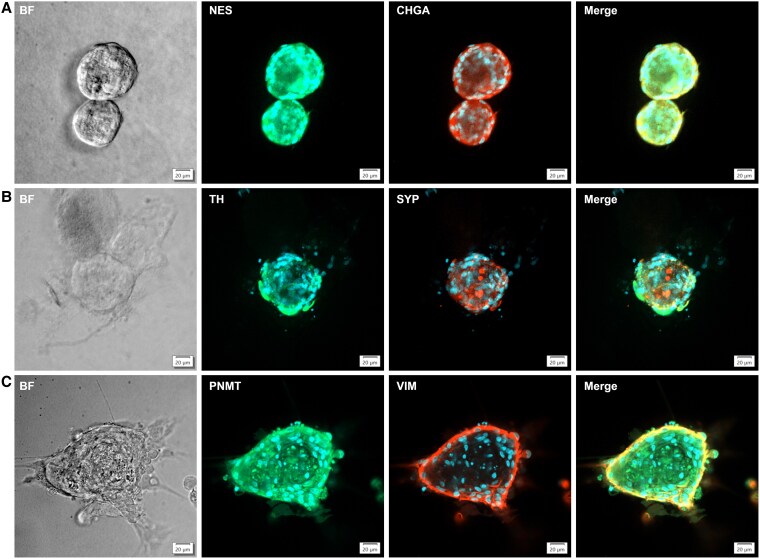
Representative immunofluorescence images of organoids derived from one adrenomedullary organoid line (NAM5), showing staining for differentiated chromaffin cell markers and progenitor/stem cell markers: NES and CHGA (A), TH and SYP (B), and PNMT and VIM (C). The specific markers are indicated at the top of each panel. Alexa Fluor 488- and Alexa Fluor 568-conjugated antibodies were used for detection, and nuclei were counterstained with DAPI. Scale bars represent 20 μm.

Abbreviations: BF, bright field; CHGA, chromogranin A; NES, nestin; PNMT, phenylethanolamine N-methyltransferase; SYP, synaptophysin; TH, tyrosine hydroxylase; VIM, vimentin.

**Figure 10.**
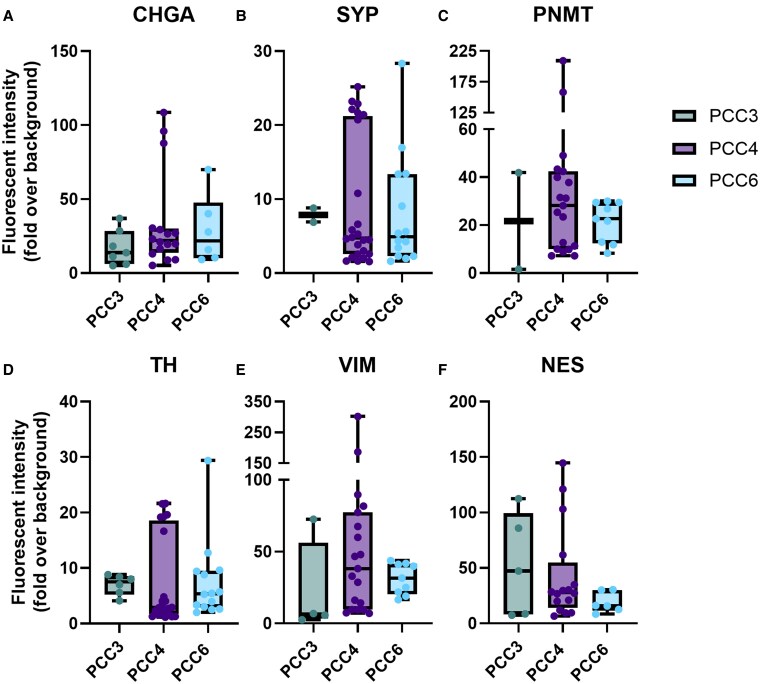
Whisker plots comparing the fold-over-background values for CHGA (A), SYP (B), PNMT (C), TH (D), VIM (E), and NES (F) in organoids derived from PCC3 (P0), PCC4 (P0), and PCC6 (P1). The fold-over-background values are expressed as the ratio of the mean fluorescence intensity of the organoid samples to the mean fluorescence intensity of the negative controls. Boxes span the interquartile range (ie, the second and third quartiles), and the line within the box represents the median fold change. Whiskers extend to the minimum and maximum values of the data, with each dot corresponding to a single organoid. The negative control organoids were exposed to the same secondary antibody combinations used for each respective staining, which consisted of Alexa Fluor™ 488 (anti-rabbit) and Alexa Fluor™ 568 (anti-mouse).

**Figure 11.**
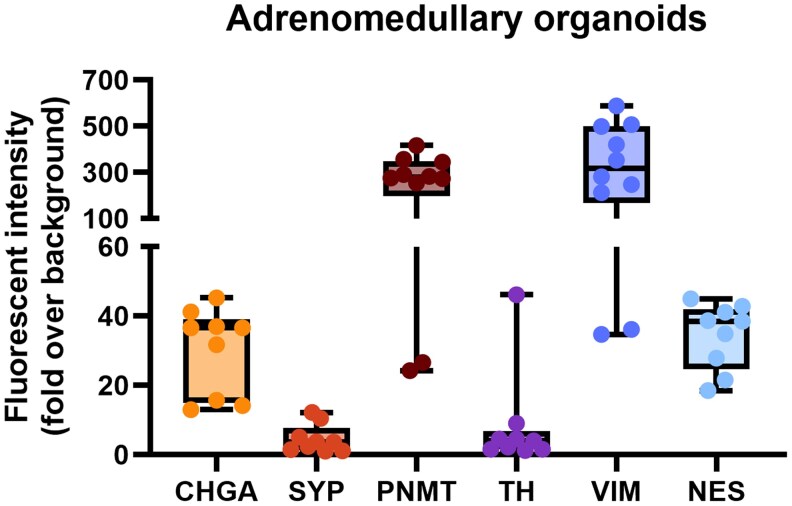
Whisker plots showing the fold-over-background values for CHGA, SYP, PNMT, TH, VIM, and NES in adrenomedullary organoids derived from NAM5 (P0). The fold-over-background values are expressed as the ratio of the mean fluorescence intensity of the organoid samples to the mean fluorescence intensity of the negative controls. Boxes span the interquartile range (ie, the second and third quartiles), and the line within the box represents the median fold change. Whiskers extend to the minimum and maximum values of the data, with each dot corresponding to a single organoid. The negative control organoids were exposed to the same secondary antibody combinations used for each respective staining, which consisted of Alexa Fluor™ 488 (anti-rabbit) and Alexa Fluor™ 568 (anti-mouse).

These immunohistochemical findings were further supported by immunofluorescence (IF) imaging, which demonstrated positive signals for the adrenomedullary markers CHGA, SYP, PNMT, and TH, as well as the stem/progenitor markers VIM and NES in both PCC and adrenomedullary organoids (Figs. 8 and 9). Fluorescence signal intensity was consistently higher than background levels, confirming the specificity of marker expression. Quantification of fluorescence intensity, expressed as fold-over-background, is shown in Figs. 10 and 11. Within PCC organoids, fluorescence intensity varied both between and within organoid lines, similar to the variability observed in IHC. This heterogeneity likely reflects differences in organoid maturation and intrinsic variability in marker expression. Notably, some cells exhibited co-expression of differentiated adrenomedullary markers and stem/progenitor markers (Figs. 8A, 8C, 9A, and 9C), suggesting that certain cells retain progenitor-like properties while expressing features of differentiated chromaffin cells under these culture conditions.

**Figure 12.**
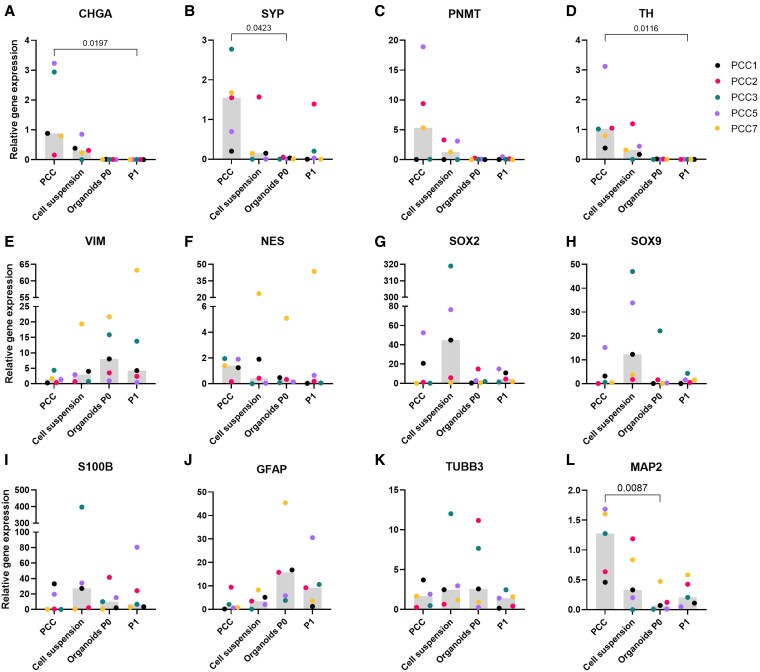
Relative gene expression levels of chromaffin cell markers *CHGA* (A), *SYP* (B), *PNMT* (C), and *TH* (D), adrenomedullary stem/progenitor markers *VIM* (E), *NES* (F), *SOX2* (G), *SOX9* (H), *S100B* (I) and *GFAP* (J), and neural markers *TUBB3* (K) and *MAP2* (L) were assessed in PCC tissues, cell suspensions, and 2 organoid passages (P0 and P1). Bars represent the median gene expression levels, while individual dots correspond to data from 5 PCCs (PCC1, PCC2, PCC3, PCC5, and PCC7). Statistical significance was determined using Friedman's test, followed by Dunn's post hoc correction for multiple comparisons. Significant differences between groups are indicated by the corresponding *P* values.

**Figure 13.**
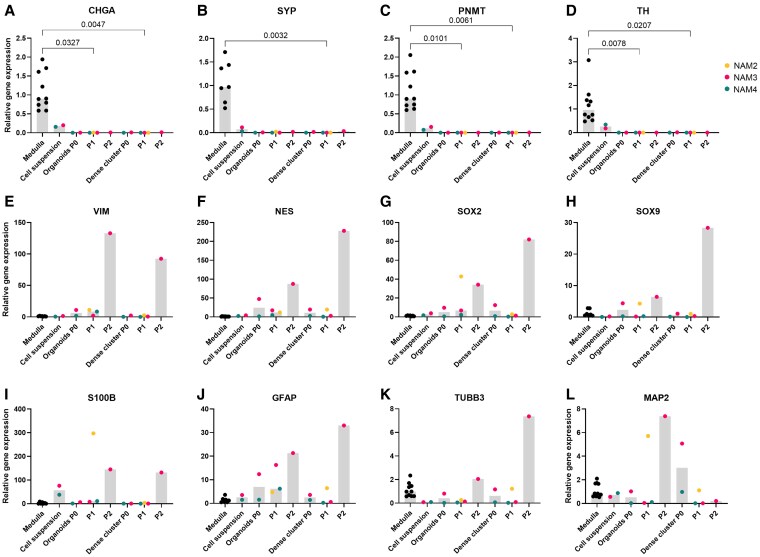
Relative gene expression levels of chromaffin cell markers *CHGA* (A), *SYP* (B), *PNMT* (C), and *TH* (D), adrenomedullary stem/progenitor markers *VIM* (E), *NES* (F), *SOX2* (G), *SOX9* (H), *S100B* (I), and *GFAP* (J), and neural markers *TUBB3* (K) and *MAP2* (L) were assessed in 3 adrenomedullary organoid lines (NAM2, NAM3, and NAM4) in various passages (P0, P1, and P2). Gene expression levels were compared to normal adrenal medulla tissue (from 7-10 samples), which were not related to the organoid lines. Bars represent the median gene expression levels, while individual dots correspond to data from the 3 adrenomedullary organoid lines. Statistical significance was determined using a Kruskal-Wallis test to compare differences between groups (normal medulla vs organoids and dense clusters across passages P0, P1, and P2), as the Friedman test could not be performed because normal medulla tissue and organoid lines were not matched. Dunn's post hoc correction for multiple comparisons was applied. Significant differences between groups are indicated by the corresponding *P* values.

The qPCR analysis provided further insights into these findings, revealing a downward trend in the mRNA expression of adrenomedullary markers (*CHGA*, *SYP*, *PNMT*, and *TH*) in PCC organoids compared to primary tumor tissue (Fig. 12), and in adrenomedullary organoids compared to NAM tissue (Fig. 13). Statistically significant lower mRNA expression levels of *CHGA* and *TH* were observed in PCC organoids at P1, and of *SYP* in PCC organoids at P0 compared to tumor tissue. For stem/progenitor markers (*VIM*, *NES*, *SOX2*, *SOX9*, *S100B*, *GFAP*), no significant differences were found between groups, although a trend toward increased mRNA expression in both adrenomedullary and PCC organoids, compared to NAM and PCC tissue, was noted. Expression levels of all markers varied substantially across different PCCs and their respective organoid lines, further highlighting the heterogeneity of the organoid cultures (Fig. 12). Gene expression analysis of PCC-derived dense cellular clusters is provided in Supplementary Fig. S6 (12).

Bright-field microscopy revealed some cells with varicose processes resembling neural structures. However, IF analysis of TUBB3 showed no significant difference in fluorescence intensity between PCC organoids and their negative controls, leading to its exclusion from further analysis. qPCR analysis revealed that *MAP2* expression was significantly lower in PCC organoids at P0 compared to PCC tissue, while *TUBB3* expression showed no significant differences (Fig. 12). Similarly, no significant differences in *TUBB3* or *MAP2* expression were observed between adrenomedullary organoids and NAM tissue (Fig. 13).

### Functional Activity of PCC Organoids: Metanephrine Concentrations

**Figure 14.**
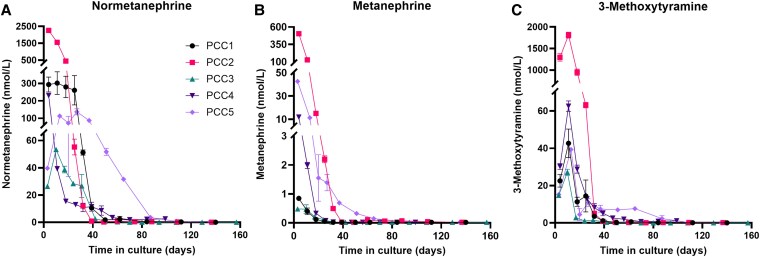
The levels of normetanephrine (A), metanephrine (B), and 3-methoxytyramine (C) in the cell culture supernatant were measured in duplicate by LC-MS/MS for 5 PCCs at different timepoints. Data points represent mean concentrations ± SD. Measurements below the limit of quantification (LOQ) were plotted at their respective LOQ values: 0.025 nmol/L for normetanephrine, 0.02 for metanephrine, and 0.01 for 3-methoxytyramine. Negative control samples showed values below the LOQ for all metanephrines.

To study the functional activity of PCC organoids, we assessed their ability to produce catecholamines, as measured by the determination of metanephrine concentrations. All organoid cultures demonstrated measurable metanephrine formation (Fig. 14), with the highest concentrations observed in passage 0 (P0). Over time, metanephrine levels declined in subsequent passages (P1 and P2). Normetanephrine levels consistently exceeded those of metanephrine across all organoid cultures, reflecting the profile observed in plasma metanephrine levels of the dogs with PCC. These findings demonstrate that organoids successfully retain key functional characteristics of the original tumors, including active catecholamine production and subsequent metanephrine formation.

### Differentiation of PCC Organoids

We attempted to induce differentiation of PCC organoids by treating them with Dex and/or PMA, with or without WREFLD. Differentiation was tested in 2 PCCs: PCC1 at P2, where a fungal infection compromised the culture, preventing any meaningful results, and PCC2 at P2, where both expansion (with WREFLD) and differentiation (with Dex or Dex + PMA, with or without WREFLD) were performed simultaneously. Among the conditions tested, WREFLD + Dex + PMA resulted in the best organoid formation, followed by WREFLD alone and WREFLD + Dex. However, the differentiation attempts were carried out during a late stage of organoid culture, when organoids were forming in small numbers and with limited size. In contrast, Dex and Dex + PMA (without WREFLD) did not clearly support organoid formation. As shown in Fig. S7 (12), metanephrine levels were measured at 2 time points for both the expansion and differentiation conditions. Although not statistically significant when compared to the WREFLD condition, an increase in metanephrine production was observed for the conditions Dex, Dex + PMA, and WREFLD + Dex + PMA.

## Discussion

In this study, we established the first adrenomedullary and PCC organoid lines, representing an important step toward developing an in vitro model that could enhance our understanding of PCC pathophysiology and support the development of novel pharmacological treatments. These organoids demonstrated self-renewal potential and expressed stem/progenitor markers such as NES and SOX10, confirming their stem/progenitor-like properties. In the early stages, organoids were able to retain differentiated chromaffin cells, as indicated by metanephrine levels in culture supernatants that initially mirrored primary tumor patterns, as well as the expression of differentiation markers such as CHGA, SYP, and PNMT. Over time, a decline in both differentiation marker expression and metanephrine levels was observed, potentially due to organoid dedifferentiation or the selective loss of differentiated chromaffin cells. These findings highlight the need for further optimization of culture conditions to sustain both proliferation and differentiation, thereby ensuring the stability and functional characteristics of the organoids over extended passages.

The adrenal medulla comprises 3 main cell types—chromaffin cells, postganglionic neurons, and supportive sustentacular cells, which recent studies suggest may also serve as progenitors (18, 24). The existence of putative adrenomedullary stem/progenitor cells in the adult adrenal medulla, capable of giving rise to new chromaffin cells, has been proposed by several studies (15, 16, 18, 19). Chromaffin progenitor cells can be enriched from adult bovine, murine, and human adrenal glands, and express a range of neural stem/progenitor markers, such as NES, VIM, Mash1, SOX9, and SOX10 (15, 16, 18). Nestin-positive progenitors can give rise to chromaffin (CHGA⁺), glial (GFAP⁺), and neuron-like (TUBB3⁺) cells, and their S100B/GFAP expression indicates their resemblance to sustentacular cells (18). Interestingly, nestin-expressing progenitor cells have also been identified in the adrenal cortex, where they migrate centripetally toward the medulla and differentiate into steroidogenic cells (25). These nestin-expressing cells in the adrenal cortex and medulla were shown to have direct interactions, highlighting the importance of cortical-medullary crosstalk in adrenal development, homeostasis, and disease (24). Recent research has further confirmed the role of sustentacular cells, showing that a subset of SOX2-expressing sustentacular cells in the adult adrenal medulla gives rise to chromaffin cells (17). Single-cell RNA sequencing revealed that these adrenomedullary stem cells also express SOX10, S100B, and GFAP, in line with previous studies.

Expression of SOX10, VIM, and NES confirms that organoids exhibit progenitor-like properties (15, 18, 26, 27). In PCC organoids, SOX10 positivity—likely reflecting sustentacular-derived progenitors—suggests a role for these cells in tumor development (28, 29). Variability in SOX10 expression between PCC organoid lines may highlight the inherent heterogeneity of PCCs and their derived organoids, with differences in cell differentiation status potentially explaining the presence of both positive and negative SOX10-expressing organoids within individual organoid lines. In contrast, NES and VIM were uniformly expressed across all organoids, consistent with findings in the adrenal medulla (18).

The expression of differentiated chromaffin markers (CHGA, SYP, PNMT, and TH) in organoids confirms the presence of chromaffin cells. However, expression varied across and within organoid lines and declined over time, likely reflecting dedifferentiation or selective loss of mature chromaffin cells (30). We observed co-expression of differentiated and stem/progenitor markers, indicating a dynamic transition between differentiation stages—consistent with reports of chromospheres (free-floating adrenomedullary progenitor aggregates) expressing both neural progenitor markers (nestin, SOX9) and CHGA, as well as progenitors transitioning toward chromaffin fates (15-17). Histological evidence of cell degeneration suggests that certain cells within the organoid cultures may be undergoing cell death. Importantly, supraphysiological oxygen tension in our cultures may have contributed to this degeneration; future studies should evaluate controlled hypoxic conditions to determine whether reduced oxygen improves long-term viability. This degeneration could contribute to the observed loss of differentiation markers. Finally, our stemness-promoting culture conditions may both induce plasticity in differentiated cells and preferentially support progenitor survival, collectively driving the loss of differentiated chromaffin marker expression over passages.

Aligned with this observation, CHGA expression was detected by IF but not by IHC in adrenomedullary organoids, likely reflecting the higher sensitivity of IF in detecting low CHGA protein levels. The reduction in metanephrine concentrations further supports a progressive loss of differentiated characteristics in the PCC organoids. We attempted to induce differentiation of PCC organoids by adding Dex and PMA to the culture media (15, 31, 32). Differentiation was attempted at a late culture stage when organoid formation had largely ceased or yielded only a few small structures, precluding meaningful IHC, qPCR, and IF analyses. Despite these challenges, we observed a non-significant increase in metanephrine production under Dex, Dex + PMA, and WREFLD + Dex + PMA conditions, suggesting an induced differentiation response. These preliminary findings require validation in larger sample sizes and extension to adrenomedullary organoids. Future work will focus on optimizing differentiation timing, factor concentrations, and screening additional compounds to promote or restore a more differentiated state in both PCC and adrenomedullary organoids over extended passages.

The WREFLD expansion cocktail combined FGF2, EGF, LIF, and DHEAS—factors known to support chromaffin cell proliferation (31, 33, 34)—with Wnt and R-spondin, which are commonly used to promote organoid formation (35). While this growth factor combination was initially effective in promoting organoid growth, its ability to sustain proliferation diminished after several passages, particularly in the PCC organoids. This decline in proliferation could be attributed to the inherently slow proliferation rates of chromaffin cells, with divisions being rare in the postnatal adrenal medulla (28), as well as in human PCCs, where doubling times can span years or even decades (3). Moreover, the variability in the self-renewal capacity of organoids could also be influenced by the genetic backgrounds of the tumors, which are highly variable in human PCC and may be similar in canine PCC. Considering this variability, a standardized approach may not be suitable for all cases, and customized growth factor combinations tailored to individual tumors may be required. Additionally, the differences in self-renewal capacity between adrenomedullary and PCC organoids may also be attributed to the fact that NAM tissue was derived from significantly younger animals, which may exhibit more robust proliferation due to age-related biological differences (33). Further research is necessary to refine culture conditions and enhance organoid growth and differentiation, including exploring co-culture with cortical cells (36), while also taking into account individual tumor characteristics.

Characterization of dense cellular clusters was limited by their compact nature—precluding IF—yet histology, IHC, and qPCR revealed profiles similar to the organoids (Figs. S1, S3, S5, and S6 (12)). These clusters resemble the fetal “adrenal organoids” described by Poli et al (2019) (37), sharing mesenchymal-like cells, nestin positivity, and extracellular matrix–rich regions. It has been hypothesized that these extracellular matrix components may play a role in coordinating spatial organization and cell turnover (37). Unlike organoids, which develop outside the BME droplet, dense clusters form as contracting aggregates within it, suggesting distinct organizational states. Further work is needed to clarify their role in organoid formation and assess their utility for disease modeling.

A notable limitation in existing PCC models is the relative scarcity of models associated with pseudohypoxia-associated cluster 1-related tumors, as nearly all identified cell lines and animal PCC models are linked to kinase signaling-associated cluster 2 tumors (2). Cluster 1 tumors, characterized by mutations in Krebs cycle genes including succinate dehydrogenase (*SDH*) subunits, von Hippel–Lindau tumor suppressor, and hypoxia-inducible factor 2α, are of particular interest as they are associated with high risk of metastasis and recurrence (4). Interestingly, *SDH* mutations have also been found in canine PCCs (8, 9), making dog-derived organoids a potentially valuable platform for studying cluster 1 biology and testing targeted therapies. Genetic characterization of the dogs in this study was not conducted, which is a significant drawback and limits the ability to fully explore the potential of this model. While one study has described the use of SDHB and succinate dehydrogenase subunit A (SDHA) IHC in dogs, these results were not validated through sequencing techniques (38). Our attempts to optimize these stainings have not been successful, aligning with findings from another study where IHC for SDHA and SDHB also proved unreliable in dogs (9). Thus, screening for *SDH* mutations in canine PCCs via IHC remains challenging, making whole genome sequencing a logical next step to identify these mutations and uncover potential additional genotypes similar to those observed in human PCC. While this approach may help further characterize canine PCCs and their derived organoids as models for the different tumor clusters, including cluster 1, we acknowledge that significant work remains to optimize and validate these models. Nevertheless, since canine PCC organoids are derived from naturally occurring tumors in a genetically heterogeneous population, and may better reflect key modulators of tumorigenesis and clinical response compared to rodent models, they hold promise as a clinically relevant model (39).

Another limitation of this study is the lack of characterization of the organoids by (single-cell) RNA sequencing, which would have provided deeper insights into their transcriptional heterogeneity and cellular composition (40).

While PCC is a rare cancer in humans, limiting the availability of tumor samples, its diagnosis in dogs is becoming increasingly common (10). With the availability of advanced medical care for veterinary patients, canine patients provide access to naturally occurring tumor samples with varying genetic backgrounds and enable both in vitro research and in vivo translational studies, with the potential to effectively bridge the gap between preclinical models and human clinical trials.

Although this study focused on establishing and characterizing canine adrenomedullary and PCC organoids, valuable initial steps have been taken toward developing an organoid platform for drug testing. Further optimization is needed to overcome challenges such as limited proliferation at later passages and differentiation before these models can be reliably applied in evaluating targeted therapies. Alongside genetic characterization of metastasis, developing metastasis-derived organoids would be valuable, as these could further enhance drug screening efforts and improve our understanding of treatment responses in advanced disease stages. Ultimately, establishing a PCC organoid biobank—comprehensively profiled by multi-omic analyses and subjected to high-throughput drug screening—could guide treatment selection by matching each new patient's tumor to molecularly matched organoid lines. Rapidly expanded patient-derived organoids would then generate individualized drug-response data to refine therapy choices (41). Organoids derived from NAM tissue could complement this approach by serving as a model for drug toxicity testing, with the potential for integration with organ-on-a-chip technology. Combining tumor-derived and normal tissue-derived organoids enables the screening for tumor-specific drug vulnerabilities while also assessing potential off-target effects, ultimately facilitating more comprehensive treatment strategies (42).

In conclusion, this study demonstrates the feasibility of establishing canine adrenomedullary and PCC organoids. While the successful generation of these organoid lines marks a significant advancement in the field, further optimization is needed to enhance their proliferation and differentiation potential, ensuring broader applicability. These organoid cultures provide a valuable foundation for future research, with the potential to advance our understanding of PCC pathophysiology and contribute to novel pharmacological treatment strategies for this challenging tumor type.

## Data Availability

Some or all datasets generated during and/or analyzed during the current study are not publicly available but are available from the corresponding author on reasonable request.

## Abbreviations

3D: three-dimensional
BME: basement membrane extract
CHGA: chromogranin A
DAPI: 4′,6-diamidino-2-phenylindole
Dex: dexamethasone
DHEAS: dehydroepiandrosterone sulfate
DMEM: Dulbecco's Modified Eagle Medium
EGF: epidermal growth factor
EM: expansion medium
FGF2: fibroblast growth factor 2
GFAP: glial fibrillary acidic protein
IF: immunofluorescence
IHC: immunohistochemistry
LC-MS/MS: liquid chromatography–tandem mass spectrometry
LIF: leukemia inhibitory factor
MAP2: microtubule-associated protein 2
NAM: normal adrenal medulla
NES: nestin
P: passage
PCC: pheochromocytoma
PMA: phorbol 12-myristate 13-acetate
PNMT: phenylethanolamine N-methyltransferase
P/S: penicillin/streptomycin
qPCR: quantitative real-time reverse transcription polymerase chain reaction
ROI: region of interest
S100B: S100 calcium-binding protein B
SDH: succinate dehydrogenase complex
SOX2: SRY-box transcription factor 2
SOX9: SRY-box transcription factor 9
SOX10: SRY-box transcription factor 10
SYP: synaptophysin
TH: tyrosine hydroxylase
TUBB3: tubulin beta 3 class III
VIM: vimentin
WREFLD: combination of growth factors Wnt, R-spondin-3, epidermal growth factor, fibroblast growth factor 2, leukemia inhibitory factor, and dehydroepiandrosterone sulfate
